# Parent Willingness to Pay for School Feeding Programs in Junior High Schools in Malang Regency, Indonesia

**DOI:** 10.3390/nu15143212

**Published:** 2023-07-19

**Authors:** Ishak Halim Octawijaya, Masahide Kondo, Ai Hori, Masao Ichikawa

**Affiliations:** 1 Graduate School of Comprehensive Human Sciences, University of Tsukuba, 1-1-1 Tennodai, Tsukuba 305-8577, Ibaraki, Japan; 2 School of Nutrition and Dietetics, Faculty of Health and Social Services, Kanagawa University of Human Services, 1-10-1 Heiseicho, Yokosuka 238-8522, Kanagawa, Japan; 3 Faculty of Medicine, University of Tsukuba, 1-1-1 Tennodai, Tsukuba 305-8577, Ibaraki, Japanmasao@md.tsukuba.ac.jp (M.I.)

**Keywords:** school feeding program, school meal, junior high school, Indonesia, willingness to pay, intention to use

## Abstract

In Indonesia, school feeding programs have not been established nationally due to the government’s limited budget. To examine the possibility of copayment for school feeding programs, parents’ intentions to use the school feeding programs and their willingness to pay (WTP) for these programs should be considered. We conducted an online questionnaire survey among the parents of junior high school students in all five public junior high schools in the Kepanjen District of Malang Regency, East Java Province, Indonesia. We used the contingent valuation method to elicit parents’ WTP for school feeding and calculated the price elasticity of school feeding. Factors associated with the WTP were examined using logistic regression analysis. Of the 940 participants, 90% intended to use school feeding programs, and 30% were willing to pay Rp 15,000 (USD 1.05) or higher per meal. Of the 944 students (participants’ children), all but two students consumed meals or snacks at school, with 74% consuming foods three or more times daily. Higher WTP for school feeding was associated with frequent food consumption at school, higher income, and a better perception of meals at school. In contrast, lower WTP was associated with more household members. Most parents intended to use school feeding programs with certain WTP irrespective of the price of school feeding. Therefore, school feeding in Indonesia might be expanded through copayment.

## 1. Introduction

School feeding programs provide health, academic, and socioeconomic benefits [1]. They facilitate a healthier diet and nutrient intake, and they reduce food insecurity among schoolchildren [2,3,4,5]. At the same time, it reduces the prevalence of anemia, underweight, and obesity [6,7,8,9]. In addition, such programs potentially enhance student attendance and academic performance [8,9,10]. In low- and middle-income countries, school feeding programs are considered strategic human capital investments; they induce healthcare expenditure reduction through their health benefits, improve future workforce productivity attributed to better academic performance, promote gender equality through female inclusion in education, and advance the local economy through the use of local resources [11,12,13].

In Indonesia, the government conducted a school feeding program called *Program Gizi Anak Sekolah* (Pro-GAS) from 2016 to 2019 [14]. Pro-GAS was initiated at 146 primary schools in two provinces, particularly in impoverished areas where stunting was prevalent. Within two years of its commencement, the central authority scaled up Pro-GAS to 632 schools in 20 provinces, and local governments were encouraged to replicate Pro-GAS in their regions [12]. However, the Special Capital Region of Jakarta was the only province that initiated a school feeding program following Pro-GAS [15]. A school feeding program review addressed that expanding Pro-GAS to the whole country is challenging, mainly because of a restrained budget [14,16].

Given the government’s limited budget, the school feeding program in Indonesia can be sustained and expanded through copayment of school feeding programs. Copayment of school feeding programs is common in high-income countries such as Japan and the United States [17,18]. However, it is uncertain whether copayment is acceptable in Indonesia, which is a middle-income country with widening economic inequality. To introduce copayment of a school feeding program, we need to understand the demand for the program and the extent of willingness to pay (WTP) for the program among its beneficiaries (i.e., parents of schoolchildren). Therefore, in the present study, we elicited their intention to use the school feeding programs and their WTP using the contingent valuation method, and we investigated factors associated with their WTP.

## 2. Materials and Methods

### 2.1. Study Setting and Participants

The study was conducted in the Kepanjen District, which is an urban area of the Malang Regency in East Java Province, Indonesia. In 2021, the population of the Malang Regency was over 2.6 million, and approximately 20% were aged below 15 years [19]. The Malang Regency had 349 junior high schools, and the school enrollment rate in 2020 was 97% [20]. Among junior high school students in East Java Province, 10% were underweight and 10% were overweight or obese in 2013 [21].

In Indonesia, schools start at 7 a.m. and end at 1 p.m., and students can have breakfast and snacks during recess at 9 a.m. and 11 a.m. During recess, it is common for students to purchase food and beverages from vendors on school premises or bring them from home to school because school feeding is not provided at junior high schools in the East Java Province.

This study’s participants included the parents (or any other guardians) of students enrolled in all five public junior high schools in the Kepanjen District in the 2021 academic year [22]. The students were in the 7th, 8th, and 9th grades (1st, 2nd, and 3rd grades of junior high school, respectively) and were between 13 and 15 years of age. Overall, there were 4386 eligible participants; they were all invited by homeroom teachers through the messaging applications the school used. Based on our pilot study’s results, to estimate the proportion of parents who were willing to pay for more than the average amount of food purchased among students at school (i.e., Rp 10,000 per meal) as approximately 0.3 to 0.5, at a 95% confidence level, a sample size of 301 to 354 was required. The sample size was calculated using OpenEpi, Version 3 [23].

### 2.2. Willingness-to-Pay

We measured the participants’ WTP for hypothetical school feeding programs using the contingent valuation method for the monetary valuation of non-market goods or services [24,25,26]. In the survey, the participants were presented with scenarios that described the attributes of hypothetical goods or services (in this case, a school feeding program), and they were requested to state their WTP or the monetary value that they were willing to pay for the goods or services presented [27]. There are several ways to elicit their WTP. In this study, we employed a payment card method, where we provided five price choices for the participants to choose the price they could pay and were willing to pay for hypothetical school feeding programs. We used four scenarios (four hypothetical school feeding programs with different attributes) as presented in the Supplementary Materials (Table S1). An example is presented in Table 1.

We developed four scenarios of hypothetical school feeding programs. Initially, we drafted the scenarios through a narrative review and expert consultation. Scenario 1 presented a school feeding program that provides 35% of the amount of vegetables and fruits recommended by the World Health Organization for a healthy diet [28]. Scenario 2 presented a school feeding program that provides vegetables and fruits under a dietitian’s supervision (menu development, nutrition calculation, and hygiene management). Scenario 3 presented a school feeding program that provides vegetables and fruits under a dietitian’s supervision, potentially decreasing students’ absence due to illness by 10% [8]. Scenario 4 (Table 1) presented a school feeding program that provides vegetables and fruits under a dietitian’s supervision, potentially decreasing students’ absence due to illness by 10% and improving students’ test scores by 5% [9].

These four scenarios were examined in focus group discussions involving 15 parents volunteering from one of the public junior high schools. We conducted four sessions of focus group discussions, with three to four participants in each session, to check the relevance of the scenarios to the study setting and to determine the appropriate range of monetary value for the hypothetical school feeding programs; these appeared to be between Rp 10,000 and 30,000 (USD 0.7 and 2.1, currency exchange rate in July 2023) per meal.

**Figure 1:**
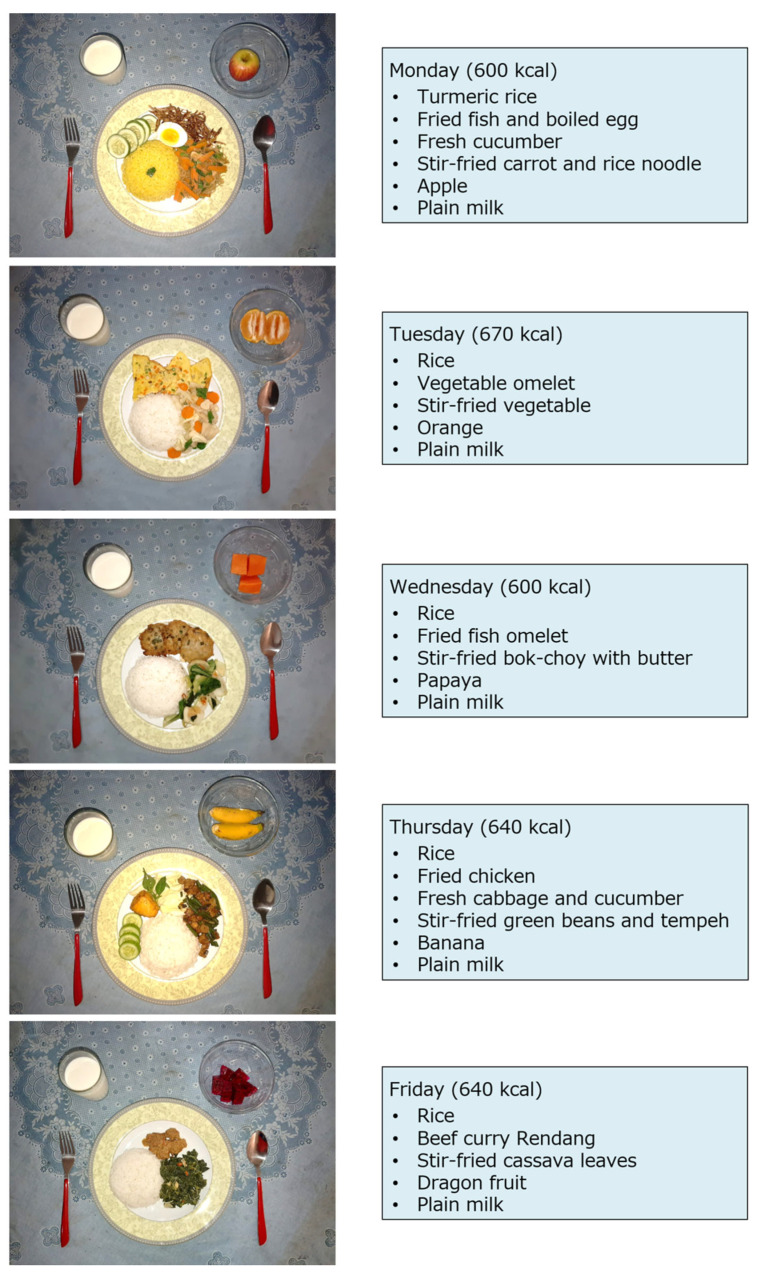
Sample Menu for the School Feeding Program Presented in the Questionnaire.

To help the participants understand the school feeding mutually, we made five different menus of local culinary dishes for five school days and presented the images to the participants (Figure 1). Each meal contained rice, a main dish (animal-based protein), a side vegetable dish, fruits, and a cup of milk. Subsequently, the revised scenarios with the images were tested in an online pilot survey, involving 23 parents volunteering from the same school to check their responses to the scenarios before being used in the main study.

The average cost per meal prepared in the present study was Rp 20,000 (USD 1.4), but this could be reduced to approximately Rp 15,000 (USD 1.05) if ingredients were purchased in the wholesale market. For comparison, the cost per meal in Pro-GAS was Rp 11,250 (USD 0.8); however, the meals in Pro-GAS provided 25% of the recommended daily allowance of food energy and protein for primary school students, whereas the meals in the present study met 30% of this daily allowance for junior high school students. The cost of both meals does not include the utility and labor costs of preparing meals.

### 2.3. Other Measures

To extract the potential factors associated with WTP for school feeding programs, we conducted a literature review regarding the factors influencing the decisions to consume healthier meal choices or the WTP for such meal choices, such as a meal with a large portion of vegetables (or other plant-based products), functional foods, or healthy school feeding. Among adolescents and adults, decisions toward healthier meal choices were commonly affected by many factors, such as food taste (or the perceptions of tastiness), personal food history (habitual food consumption), familiarity with the meal, peers, convenience (effort required to prepare it), price, perceived animal welfare, health benefits, food sensory elements (food appearance, smell, and accessibility), freshness, and dining environment [29,30,31]. Additionally, food choices among adults or younger people are altered by food allergies [32,33]. Regarding school feeding programs, customer satisfaction with service quality influences their willingness to use the programs [34]. Higher educational attainment and the health information of the product, among several other factors, are considered to drive the higher intention to use as well as higher WTP for healthier meal choices [35,36]. These characteristics and multidimensional factors are related to decisions on healthier meal choices and thus influence the amount of money people are willing to spend on healthier meal choices, including healthy school feeding programs.

Therefore, we collected the following information. Data on the participants’ characteristics included: age; relationship with student; educational attainment; occupation; number of household members; household monthly income (before and after the COVID-19 pandemic); recognition and previous use of school feeding; perception of meals at school relative to meals at home, in terms of healthiness, taste, and convenience; satisfaction with meals at school; and intention to use hypothetical school feeding programs. The participants’ characteristics are presented in Table 2. Data on their children’s characteristics included: age, sex, ethnicity, religion, food restriction, monthly food budget to purchase foods and drinks at school, and frequency of meals and snacks consumed at home and school before the pandemic.

Perception of meals at school relative to meals at home, in terms of healthiness, taste, and convenience, was rated on a 5-point Likert scale. These included: “meals at home are better than those at school”, “meals at home are slightly better than those at school”, “both are equal”, “meals at school are slightly better than those at home”, and “meals at school are better than those at home”. Lastly, satisfaction with meals at school and intention to use hypothetical school feeding programs were measured using a 4-point Likert scale: “satisfied”, “slightly satisfied”, “slightly unsatisfied”, and “unsatisfied”; and “I will certainly use”, “I might use”, “I might not use”, and “I will certainly not use”. Along with the four contingent valuation scenarios, these variables were included in the questionnaire and piloted during the focus group discussions and the online pilot survey.

The participants were instructed to answer the questions with their children, separately for each child if they had two or more children in the same school. Participants with 7th graders (first-year junior high school students) who did not attend junior high school because of the pandemic were asked to answer based on their experiences in primary school. Like those in junior high schools, students in primary school are allowed to purchase and consume food and beverages during recess.

### 2.4. Data Collection

We collected data anonymously using Google Forms (https://docs.google.com/forms/, last accessed 15 July 2023) from January to March 2021. To request all eligible participants to participate in this online survey, their children’s schools sent them a link to our Google Forms with a letter of request via messaging applications used for communication between the schools and the guardians. Those who agreed to participate in the survey were requested to answer the questions using Google Forms.

This study was conducted in accordance with the guidelines of the Declaration of Helsinki, and all procedures involving research study participants were approved by the Faculty of Medicine at the University of Tsukuba in Japan (No. 1583) and the local authority of Malang Regency in Indonesia. Parents were explained that the study aimed to elicit parents’ opinions regarding school feeding programs if introduced in their child’s school. We mentioned that their opinions would be helpful and accounted for when we expand school feeding programs such as Pro-GAS, which provide academic, economic, health, and social benefits for students and the local community. We provided the participants of focus group discussions with small souvenirs but did not do so for the other survey participants as the surveys were anonymous and conducted online.

### 2.5. Statistical Analyses

First, we described the participants’ characteristics, perceptions of meals at school relative to that of meals at home, and their intention to use school feeding programs. Thereafter, we graphed an inverse demand curve based on the parents’ WTP for school feeding programs in each scenario to examine the price elasticity of school feeding (i.e., the extent to which the price would influence the demand for school feeding). More precisely, the inverse demand curve expresses the relationship between the price per meal in the school feeding programs and the quantity or number of participants demanding the school feeding program in natural log form. The coefficient of the log quantity of the school feeding program demanded in the inverse demand curve represents the ratio of the percentage change in quantity to the percentage change in price, which is the price elasticity of school feeding programs [37]. A ratio < 1 indicates that the price of school feeding programs is inelastic (i.e., the lower the ratio, the lower the price that would influence demand for the programs).

Finally, we conducted logistic regression analyses using IBM SPSS Statistics 24 to investigate the factors associated with WTP for the school feeding program. The WTP of each participant (either Rp 10,000, Rp 15,000, Rp 20,000, Rp 25,000, or Rp 30,000) was dichotomized into Rp 10,000 per meal and Rp 15,000 or higher per meal, considering the average food purchase of Rp 10,000 per day among junior high school students in our previous study but in a different city in Indonesia [38]. The WTP for each scenario is presented in Table 3. Potential factors associated with the WTP included in the analysis were students’ food restriction, frequency of food consumption at school, household income during the pandemic, number of household members, educational attainment, perception of meals at school relative to meals at home, satisfaction with meals at school, and recognition and previous use of school feeding programs [29,30,32,33,34,35,36,39]. The categories of the variables are listed in Table 4. We repeated the same analysis for the four scenarios, and the findings appeared consistent (Supplementary Materials Table S2). Therefore, we present the findings for Scenario 4 in the Results section. Note that the analysis did not consider a multilevel data structure of children within a family because only four of the 940 participants had two children in the same school.

In this study, all statistical analyses were conducted based on the hypotheses and methodology planned before the data collection. Data-driven analyses were identified and discussed appropriately.

## 3. Results

### 3.1. Participants’ Characteristics

Of the 4386 eligible participants, 940 (22%) participated in the online survey (Table 2). The 940 participants were aged 42 years on average, 74% were mothers, and 75% had attained up to secondary education. The median household size had four members and the median household monthly income before and during the pandemic was Rp 2,500,000 (USD 175) and Rp 2,000,000 (USD 140), respectively. While school feeding was not provided in the study area, 38% of the participants recognized that school feeding programs had been implemented in the country, and 24% of them had used some form of school feedings, such as school-managed catering services and government-led school feeding programs, probably during their childhood. Regarding their perception of meals at school, relative to meals at home, many participants perceived that meals at home were healthier (73%), tastier (60%), and more convenient (56%) than meals at school. Nevertheless, most participants were satisfied with meals at school (92%) and intended to use the school feeding program proposed in this study (91%).

Regarding the characteristics of the participants’ children (i.e., 944 students), 56% were female, 38%, 31%, and 31% were in the 7th, 8th, and 9th grades, respectively; 98% were Javanese, 96% were Muslim, and 10% had food allergies. All but two students consumed foods or beverages at school, with 33% consuming three times per day and 41% consuming four times or more per day (not shown in the table).

### 3.2. Willingness to Pay for The School Feeding Program

**Figure 2:**
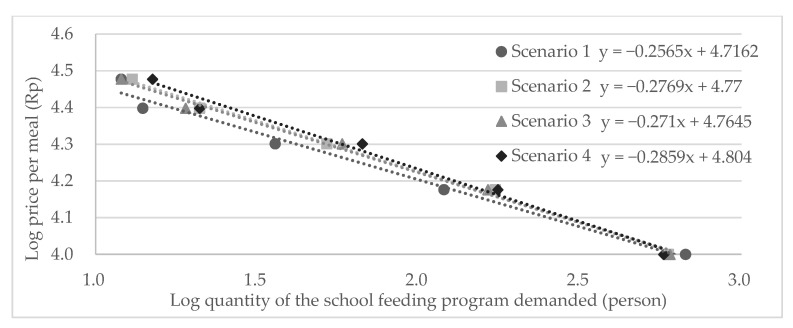
The Inverse Demand Curve for the School Feeding Programs.

Across the four scenarios, 21–31% of the participants were willing to pay Rp 15,000 or higher per meal (Table 3). The more the benefits presented in the hypothetical school feeding program, the higher the WTP elicited. In all scenarios, the price elasticity of the school feeding program is approximately 0.3; thus, it is inelastic (Figure 2).

### 3.3. Factors Associated with Willingness to Pay

Participants were more likely to pay Rp 15,000 or higher per meal if they perceived the meals at school as healthier than those at home, if their children consumed foods or drinks at school three or more times a day, or if they had a higher income (Table 4). Conversely, those with more household members were less likely to pay Rp 15,000 or more per meal.

## 4. Discussion

School feeding programs were in high demand among parents of junior high school students: 90% of the study participants intended to use school feeding programs. This is consistent with the inelasticity in the WTP for hypothesized school feeding, indicating that school feeding is a necessity for parents and schoolchildren (i.e., the demand would be stable irrespective of the price of school feeding). However, parents with more household members or a lower household income had a lower WTP for school feeding.

While the hypothesized school feeding programs were widely accepted, 70% of the participants chose the lowest price of Rp 10,000 per meal in contingent valuation. This could be partly due to the bias caused by the price range. Alternatively, they might have considered Rp 10,000 appropriate for school feeding because this is the average amount of students’ daily spending on food and beverages at school [38]. In contrast, 10% of participants did not intend to use school feeding programs. In the survey, the benefits of school feeding were explained fully to the participants. It is unclear whether the participants who let their children bring meals from home to school perceived the benefits as not sufficiently attractive or whether they considered school feeding as unnecessary.

To expand school feeding programs in Indonesia, measures must be taken to cover the cost of these programs, including not only food costs but also utility costs, labor costs, and other additional costs. The expected food cost per meal of the hypothesized school feeding was Rp 15,000, and 30% of the participants were willing to pay this price or higher. If the food cost could be reduced, it might be covered by the amount paid by the beneficiaries. For the full recovery of other costs, the central or local authority might allocate a budget. In fact, some local authorities provide subsidies for school feeding in Indonesia [15]. Considering that participants with a lower income had a lower WTP, the authorities should consider offering discounts for students from impoverished households to avail school feeding to all students [17,18].

This study had two major limitations. First, participants’ responses to WTP for school feeding might have been influenced by the payment card method in contingent valuation. We used this method because it was straightforward for the participants to answer through an online survey, in which they were asked to choose one of several price choices. However, the drawback of this method is that their responses can be influenced by the price range presented to them. To avoid such a range bias, we have conducted focus group discussions to determine an appropriate price range.

Second, the participants’ intention to use and the WTP for school feeding programs might have been overestimated if they were more interested in school feeding than non-participants. It should be noted that only 22% of invited parents participated in this study; among these participants, 25% had higher education, whereas this proportion was 5% among people over the age of 15 in Malang Regency [40]. Their educational attainment might have resulted in their interest in school feeding, although it was not associated with their WTP. Further investigation among a representative sample of parents in the region is needed before introducing school feeding and copayment for school feeding.

In conclusion, copayment could be a viable option for expanding school feeding programs in Indonesia. This study revealed that many parents intended to use and are willing to pay for the food cost of school feeding, indicating that copayment could be a sound solution for establishing these programs. By implementing copayment, schools could offer students more nutritious meals and improve their communities’ overall health and well-being.

## Figures and Tables

**Table 1:** An Example of the Scenarios Presented in the Study (Scenario 4).

Suppose the school terminates the current school food system and starts a new school feeding program for all students that will replace breakfast at home. You may choose a new kind of nutrition management of the program at extra cost. The next few questions will ask about how much extra cost you would be prepared to pay for different types of nutrition management. Please consider how much you can actually afford.				
Scenario 4 In addition to health benefits of the school feeding program managed by a dietitian, you could choose to have the program to decrease student absence due to illness by 10% and improve student’s test scores by 5%. Considering how much you can afford monthly (20 school days/month), what is the most that you would be prepared to pay to have this health feature fitted per meal?				
Rp 10,000 (Rp 200,000/month)	Rp 15,000 (Rp 300,000/month)	Rp 20,000 (Rp 400,000/month)	Rp 25,000 (Rp 500,000/month)	Rp 30,000 (Rp 600,000/month)

**Table 2:** Participants’ Characteristics, Perception of Meals at School Relative to Meals at Home, and Intention to Use School Feeding Programs.

Variables	*n*	%
Age, mean (SD)	41.6	(7.5)
Relationship with student		
Mother	693	73.7
Father	203	21.6
Others such as older siblings and grandparents	44	4.7
Educational attainment		
Secondary school/lower	700	74.5
Undergraduate/higher	240	25.5
Number of household members, median (IQR)	4	(4, 5)
Parent occupation before pandemic		
Unemployed/unpaid family worker	326	34.5
Freelancer	53	5.6
Laborer/worker	325	34.4
Entrepreneur without worker	175	18.5
Employer with temporary/permanent laborer/worker	65	6.9
Parent occupation after pandemic		
Unemployed/unpaid family worker	337	35.7
Freelancer	54	5.7
Laborer/worker	297	31.5
Entrepreneur without worker	193	20.4
Employer with temporary/permanent laborer/worker	63	6.7
Household income before the pandemic (Rp), median (IQR)	2,500,000	(1,500,000, 4,000,000)
Household income during the pandemic (Rp), median (IQR)	2,000,000	(1,000,000, 3,000,000)
Healthiness of meals at school relative to meals at home		
Meals at home are better	686	73.0
Equal	232	24.7
Meals at school are better	22	2.3
Taste of meals at school relative to meals at home		
Meals at home are better	567	60.3
Equal	212	22.6
Meals at school are better	161	17.1
Convenience of meals at school relative to meals at home		
Meals at home are better	530	56.4
Equal	265	28.2
Meals at school are better	145	15.4
Satisfaction with meals at school		
Satisfied	433	46.1
Slightly satisfied	431	45.9
Slightly unsatisfied	73	7.8
Unsatisfied	3	0.3
Intention to use hypothetical school feeding programs		
I will certainly use	370	39.0
I might use	480	51.1
I might not use	50	5.3
I will certainly not use	40	4.3

**Table 3:** Parents’ Willingness to Pay for Four Hypothetical School Feeding Programs.

Price Per Meal	Scenario 1 ^a^		Scenario 2 ^b^		Scenario 3 ^c^		Scenario 4 ^d^	
	*n*	%	*n*	%	*n*	%	*n*	%
Rp 10,000	747	79	669	71	676	72	649	69
Rp 15,000	129	14	182	19	171	18	184	19
Rp 20,000	37	4	54	6	62	7	70	7
Rp 25,000	16	2	23	2	20	2	22	2
Rp 30,000	15	2	16	2	15	2	19	2

^a^ Scenario 1: WTP for school feeding that provides vegetables and fruits as much as 35% of the intake amount advised by WHO. ^b^ Scenario 2: WTP for school feeding that provides vegetables and fruits with a dietitian’s supervision (menu development, nutrition calculation, and hygiene management). ^c^ Scenario 3: WTP for school feeding that provides vegetables and fruits with a dietitian’s supervision, which has the potential to decrease student absence due to illness by 10%. ^d^ Scenario 4: WTP for school feeding that provides vegetables and fruits with a dietitian’s supervision, which has the potential to decrease student absence due to illness by 10% and improve students’ test score by 5%.

**Table 4:** Factors Associated with Parents’ Willingness-to-Pay for the School Feeding Program.

Variables	Number of Participants	≧Rp 15,000 per Meal	Unadjusted		Adjusted	
		*n* (%)	OR	(95% CI)	OR	(95% CI)
Educational attainment						
Secondary school or lower	704	212 (30%)				
Undergraduate or higher	240	83 (35%)	1.2	0.9–1.7	1.2	0.8–1.6
Number of household members						
Less than 4	157	60 (38%)				
4 persons (median)	349	108 (31%)	0.7	0.5–1.1	0.7	0.5–1.1
More than 4	438	127 (29%)	0.7 *	0.5–1.0	0.6 *	0.4–0.9
Household income during the pandemic						
Lower income (T1)	301	79 (26%)				
Middle income (T2)	300	92 (31%)	1.2	0.9–1.8	1.2	0.8–1.8
Higher income (T3)	343	124 (36%)	1.6 **	1.1–2.2	1.6 **	1.1–2.4
Healthiness of meals at school relative to meals at home						
Meals at home are better	690	221 (32%)				
Equal	232	62 (27%)	0.8	0.6–1.1	0.8	0.5–1.2
Meals at school are better	22	12 (55%)	2.5 *	1.1–6.0	2.8 *	1.1–6.7
Taste of meals at school relative to meals at home						
Meals at home are better	570	185 (32%)				
Equal	212	65 (31%)	0.9	0.7–1.3	0.9	0.7–1.4
Meals at school are better	162	45 (28%)	0.8	0.5–1.2	0.7	0.5–1.0
Convenience of meals at school relative to meals at home						
Meals at home are better	534	166 (31%)				
Equal	265	79 (30%)	0.9	0.7–1.3	1.0	0.7–1.4
Meals at school are better	145	50 (34%)	1.2	0.8–1.7	1.2	0.8–1.9
Satisfaction with meals at school						
Unsatisfied	76	24 (32%)				
Satisfied	868	271 (31%)	1.0	0.6–1.6	0.9	0.5–1.6
Recognized any school feeding program previously						
No	577	176 (31%)				
Yes	367	119 (32%)	1.1	0.8–1.4	1.2	0.8–1.7
Experience in using any school feeding program						
Never	713	228 (32%)				
Yes	231	67 (29%)	0.9	0.6–1.2	0.8	0.5–1.1
Students having food restriction						
No	848	261 (31%)				
Yes	96	34 (35%)	1.2	0.8–1.9	1.2	0.8–1.9
Frequency of food consumption at school before the pandemic						
Less than 3 times/day	244	54 (22%)				
3 times/day	309	111 (36%)	2.0 **	1.3–2.9	2.1 **	1.4–3.2
4 times/day or more	391	130 (33%)	1.8 **	1.2–2.5	2.0 **	1.3–2.9

* *p* < 0.05; ** *p* < 0.01.

## Data Availability

The data presented in this study are available on request from the corresponding author.

## Supplementary Materials

The following supporting information can be downloaded at: https://www.mdpi.com/article/10.3390/nu15143212/s1, Table S1: The Scenarios Presented in the Study (Scenario 1–4); Table S2: Factors Associated with Parents’ Willingness-to-Pay for the School Feeding Program (Scenario 1–4).
