# Supplementary material for: Parent Willingness to Pay for School Feeding Programs in Junior High Schools in Malang Regency, Indonesia
Source: Nutrients. 2023 Jul 19;15(14):3212. doi: 10.3390/nu15143212 (PMC10384078; doi:10.3390/nu15143212)
Supplement: Supplementary file 1 [file nutrients-15-03212-s001.zip › nutrients-2475291-supplementary.pdf]

Article

Parent Willingness to Pay for School Feeding Programs in Junior High Schools in Malang Regency, Indonesia

Supplementary Materials

Table S1. The Scenarios Presented in The Study (Scenario 1-4)

Suppose the school terminates the current school food system and starts a new school feeding program for all students that will replace breakfast at home. You may choose a new kind of nutrition management of the program at extra cost. The next few questions will ask about how much extra cost you would be prepared to pay for different types of nutrition management. Please consider how much you can actually afford.

High intake of sugar, salt, and saturated fat along with low intake of vegetables and fruit is a risk factor of chronic diseases such as heart disease, stroke, and some cancers (stomach, esophageal, lung, and colorectal cancers). Now, 96.8% of 10-14 years old children in Indonesia consume vegetables and fruit less than the recommended level.

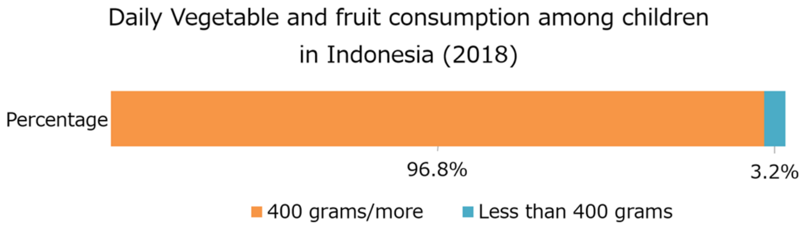

Scenario 1

You could choose to have a nutrient standard applied to breakfast program which will provide vegetable and fruit as much as 35% of recommended daily intake (see graph below). Taking into account how much you can afford monthly (20 school days/month), what is the most that you would be prepared to pay to have this health feature fitted per meal?

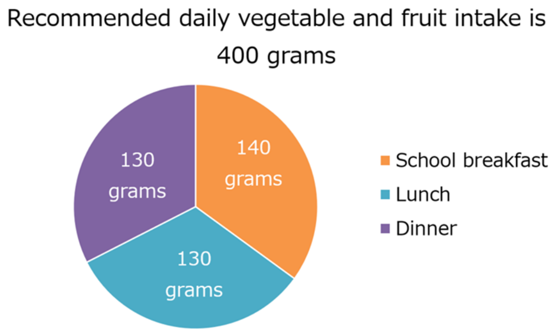

Rp 10,000 (Rp 200,000/ month)	Rp 15,000 (Rp 300,000/ month)	Rp 20,000 (Rp 400,000/ month)	Rp 25,000 (Rp 500,000/ month)	Rp 30,000 (Rp 600,000/ month)

Scenario 2

An alternative nutrient standard combined with dietitian supervision applied to breakfast program will provide vegetable and fruit as much as 35% of recommended daily intake (see graph below). Taking into account how much you can afford monthly (20 school days/month), what is the most that you would be prepared to pay to have this health feature fitted per meal?

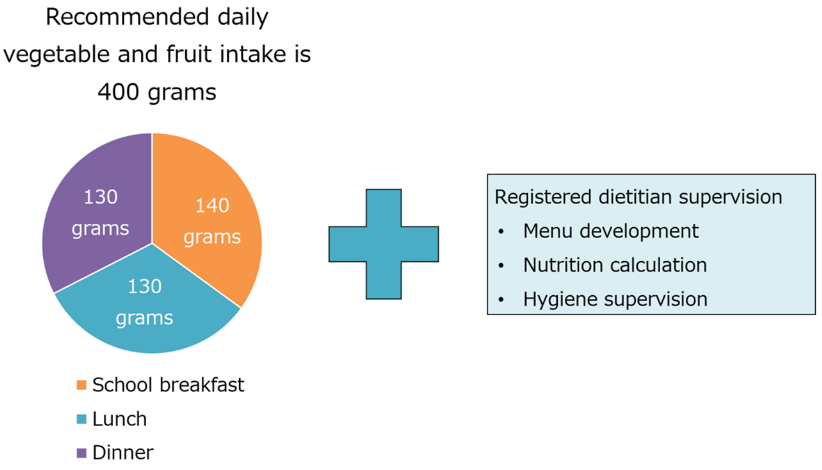

Rp 10,000 (Rp 200,000/ month)	Rp 15,000 (Rp 300,000/ month)	Rp 20,000 (Rp 400,000/ month)	Rp 25,000 (Rp 500,000/ month)	Rp 30,000 (Rp 600,000/ month)

Scenario 3

In addition to health benefits of school feeding program, it also enhances student attendance and academic performance at school [Frisvold 2015] [WFP 2016] [WFP 2018]. You could choose to have a breakfast program to decrease student absence due to illness by 10%. Taking into account how much you can afford monthly (20 school days/month), what is the most that you would be prepared to pay to have this health feature fitted per meal?

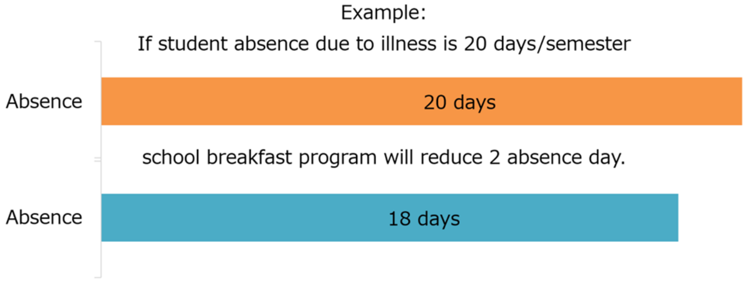

Rp 10,000 (Rp 200,000/ month)	Rp 15,000 (Rp 300,000/ month)	Rp 20,000 (Rp 400,000/ month)	Rp 25,000 (Rp 500,000/ month)	Rp 30,000 (Rp 600,000/ month)

Scenario 4

In addition to health benefits of the school feeding program managed by a dietitian, you could choose to have the program to decrease student absence due to illness by 10% and improve student's test scores by 5%. Considering how much you can afford monthly (20 school days/month), what is the most that you would be prepared to pay to have this health feature fitted per meal?

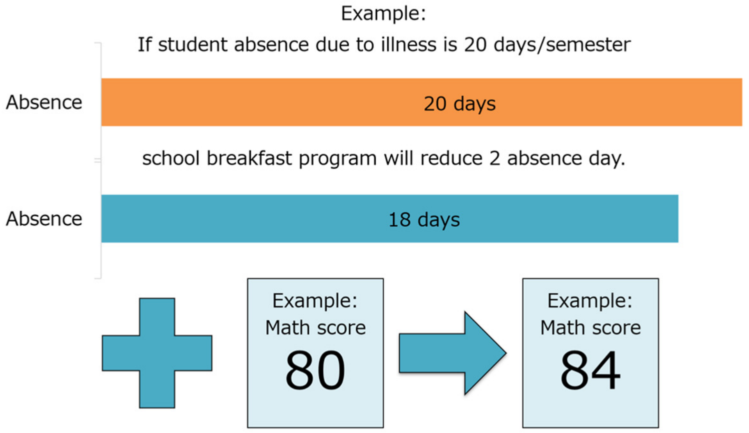

Rp 10,000 (Rp 200,000/ month)	Rp 15,000 (Rp 300,000/ month)	Rp 20,000 (Rp 400,000/ month)	Rp 25,000 (Rp 500,000/ month)	Rp 30,000 (Rp 600,000/ month)

Table S2. Factors Associated with Parents' Willingness-to-Pay for The School Feeding Program (Scenario 1-4)

Variables	Number of participants	Scenario 1					Scenario 2				
		≧Rp 15,000 per meal	Unadjusted		Adjusted		≧Rp 15,000 per meal	Unadjusted		Adjusted	
		n (%)	OR	(95% CI)	OR	(95% CI)	n (%)	OR	(95% CI)	OR	(95% CI)
Educational attainment											
Secondary school or lower	704	141 (20%)					190 (27%)				
Undergraduate or higher	240	56 (23%)	1.2	0.9-1.7	1.2	0.8-1.8	85 (35%)	1.5	1.1-2.0	1.4*	1.0-2.0
Number of household members											
Less than 4	157	40 (25%)					60 (38%)				
4 persons (median)	349	72 (21%)	0.8	0.5-1.2	0.7	0.5-1.2	99 (28%)	0.6	0.4-1.0	0.6*	0.4-0.9
More than 4	438	85 (19%)	0.7	0.5-1.1	0.7	0.4-1.1	116 (26%)	0.6**	0.4-0.9	0.5**	0.4-0.8
Household income during the pandemic*											
Lower income (T1)	301	52 (17%)					76 (25%)				
Middle income (T2)	300	56 (19%)	1.1	0.7-1.7	1.1	0.7-1.7	79 (26%)	1.1	0.7-1.5	1.0	0.7-1.5
Higher income (T3)	343	89 (26%)	1.7**	1.1-2.5	1.8**	1.2-2.7	120 (35%)	1.6**	1.1-2.2	1.6*	1.1-2.3
Healthiness of meals at school relative to meals at home											
Meals at home are better	690	143 (21%)					200 (29%)				
Equal	232	46 (20%)	0.9	0.7-1.4	1.0	0.7-1.6	62 (27%)	0.9	0.6-1.2	0.9	0.6-1.4
Meals at school are better	22	8 (36%)	2.2*	0.9-5.3	2.3	0.9-5.8	13 (59%)	3.5**	1.5-8.4	4.3**	1.7-10.6
Taste of meals at school relative to meals at home											
Meals at home are better	570	120 (21%)					175 (31%)				
Equal	212	42 (20%)	0.9	0.6-1.4	0.9	0.6-1.4	56 (26%)	0.8	0.6-1.2	0.8	0.5-1.1
Meals at school are better	162	35 (22%)	1.0	0.7-1.6	0.9	0.6-1.4	44 (27%)	0.8	0.6-1.2	0.7	0.5-1.1
Convenience of meals at school relative to meals at home											
Meals at home are better	534	110 (21%)					147 (28%)				
Equal	265	56 (21%)	1.0	0.7-1.5	1.0	0.7-1.5	80 (30%)	1.1	0.8-1.6	1.3	0.9-1.8
Meals at school are better	145	31 (21%)	1.0	0.7-1.6	1.1	0.7-1.7	48 (33%)	1.3	0.9-1.9	1.4	0.9-2.1
Satisfaction with meals at school											
Unsatisfied	76	15 (20%)					21 (28%)				
Satisfied	868	182 (21%)	1.1	0.6-1.9	1.0	0.6-1.9	254 (29%)	1.1	0.6-1.8	1.0	0.6-1.8
Recognized any school feeding program previously											
No	577	126 (22%)					168 (29%)				
Yes	367	71 (19%)	0.9	0.6-1.2	0.9	0.6-1.4	107 (29%)	1.0	0.8-1.3	1.0	0.7-1.4
Experience in using any school feeding program											
Never	713	157 (22%)					211 (30%)				
Yes	231	40 (17%)	0.7	0.5-1.1	0.7	0.5-1.2	64 (28%)	0.9	0.7-1.3	0.9	0.6-1.3
Students having food restriction											
No	848	167 (20%)					242 (29%)				
Yes	96	30 (31%)	1.9**	1.2-2.9	1.8*	1.1-2.9	33 (34%)	1.3	0.8-2.1	1.3	0.8-2.1
Frequency of food consumption at school before the pandemic											
Less than 3 times/day	244	30 (12%)					57 (23%)				
3 times/day	309	73 (24%)	2.2**	1.4-3.5	2.3**	1.4-3.8	97 (31%)	1.5	1.0-2.2	1.6*	1.1-2.4
4 times/day or more	391	94 (24%)	2.3**	1.4-3.5	2.5**	1.6-3.9	121 (31%)	1.5	1.0-2.1	1.6*	1.1-2.4

Variables	Number of participants	Scenario 3					Scenario 4				
		≧Rp 15,000 per meal	Unadjusted		Adjusted		≧Rp 15,000 per meal	Unadjusted		Adjusted	
		n (%)	OR	(95% CI)	OR	(95% CI)	n (%)	OR	(95% CI)	OR	(95% CI)
Educational attainment											
Secondary school or lower	704	185 (26%)					212 (30%)				
Undergraduate or higher	240	83 (35%)	1.5	1.1-2.0	1.4	1.0-2.0	83 (35%)	1.2	0.9-1.7	1.2	0.8-1.6
Number of household members											
Less than 4	157	51 (32%)					60 (38%)				
4 persons (median)	349	92 (26%)	0.7	0.5-1.1	0.7	0.5-1.1	108 (31%)	0.7	0.5-1.1	0.7	0.5-1.1
More than 4	438	125 (29%)	0.8*	0.6-1.2	0.8	0.5-1.2	127 (29%)	0.7*	0.5-1.0	0.6*	0.4-0.9
Household income during the pandemic*											
Lower income (T1)	301	72 (24%)					79 (26%)				
Middle income (T2)	300	81 (27%)	1.2	0.8-1.7	1.2	0.8-1.7	92 (31%)	1.2	0.9-1.8	1.2	0.9-1.8
Higher income (T3)	343	115 (34%)	1.6**	1.1-2.3	1.5*	1.1-2.3	124 (36%)	1.6**	1.1-2.2	1.6**	1.1-2.4
Healthiness of meals at school relative to meals at home											
Meals at home are better	690	199 (29%)					221 (32%)				
Equal	232	59 (25%)	0.8	0.6-1.2	0.9	0.6-1.4	62 (27%)	0.8	0.6-1.1	0.8	0.5-1.2
Meals at school are better	22	10 (45%)	2.1*	0.9-4.8	2.3	0.9-5.6	12 (55%)	2.5*	1.1-6.0	2.8*	1.1-6.7
Taste of meals at school relative to meals at home											
Meals at home are better	570	170 (30%)					185 (32%)				
Equal	212	57 (27%)	0.9	0.6-1.2	0.9	0.6-1.3	65 (31%)	0.9	0.7-1.3	0.9	0.7-1.4
Meals at school are better	162	41 (25%)	0.8	0.5-1.2	0.7	0.5-1.1	45 (28%)	0.8	0.5-1.2	0.7	0.5-1.0
Convenience of meals at school relative to meals at home											
Meals at home are better	534	150 (28%)					166 (31%)				
Equal	265	72 (27%)	1.0	0.7-1.3	1.0	0.7-1.5	79 (30%)	0.9	0.7-1.3	1.0	0.7-1.4
Meals at school are better	145	46 (32%)	1.2	0.8-1.8	1.3	0.8-2.0	50 (34%)	1.2	0.8-1.7	1.2	0.8-1.9
Satisfaction with meals at school											
Unsatisfied	76	24 (32%)					24 (32%)				
Satisfied	868	244 (28%)	0.8	0.5-1.4	0.8	0.5-1.4	271 (31%)	1.0	0.6-1.6	0.9	0.5-1.6
Recognized any school feeding program previously											
No	577	162 (28%)					176 (31%)				
Yes	367	106 (29%)	1.0	0.8-1.4	1.0	0.7-1.5	119 (32%)	1.1	0.8-1.4	1.2	0.8-1.7
Experience in using any school feeding program											
Never	713	204 (29%)					228 (32%)				
Yes	231	64 (28%)	1.0	0.7-1.3	0.9	0.6-1.4	67 (29%)	0.9	0.6-1.2	0.8	0.5-1.1
Students having food restriction											
No	848	236 (28%)					261 (31%)				
Yes	96	32 (33%)	1.3	0.8-2.0	1.3	0.8-2.1	34 (35%)	1.2	0.8-1.9	1.2	0.8-1.9
Frequency of food consumption at school before the pandemic											
Less than 3 times/day	244	54 (22%)					54 (22%)				
3 times/day	309	87 (28%)	1.4	0.9-2.0	1.5*	1.0-2.3	111 (36%)	2.0**	1.3-2.9	2.2**	1.4-3.2
4 times/day or more	391	127 (32%)	1.7**	1.2-2.4	1.9**	1.3-2.8	130 (33%)	1.8**	1.2-2.5	2.0**	1.3-2.9
